# Clinical Significance of Circulating Tumor Cell Induced Epithelial-Mesenchymal Transition in Patients with Metastatic Colorectal Cancer by Single-Cell RNA-Sequencing

**DOI:** 10.3390/cancers13194862

**Published:** 2021-09-28

**Authors:** Masahiro Kozuka, Francesca Battaglin, Priya Jayachandran, Jingyuan Wang, Hiroyuki Arai, Shivani Soni, Wu Zhang, Mitsuharu Hirai, Satoshi Matsusaka, Heinz-Josef Lenz

**Affiliations:** 1 Division of Medical Oncology, Norris Comprehensive Cancer Center, Keck School of Medicine, University of Southern California, Los Angeles, CA 90033, USA; fbattagl@usc.edu (F.B.); Priya.Jayachandran@med.usc.edu (P.J.); 1811110576@bjmu.edu.cn (J.W.); hiroyuki.arai.1217@gmail.com (H.A.); ss_558@usc.edu (S.S.); Wu.Zhang@med.usc.edu (W.Z.); 2 Research and Development Division, ARKRAY, Inc., Kyoto 602-0008, Japan; hiraim@arkray.co.jp; 3 Department of Clinical Research and Regional Innovation, Faculty of Medicine, University of Tsukuba, Tsukuba 305-8576, Japan; matsusaka-s@md.tsukuba.ac.jp; 4 Tsukuba Clinical Research and Development Organization, University of Tsukuba, Tsukuba 305-8575, Japan

**Keywords:** circulating tumor cell, single-cell, RNA-sequencing, epithelial-mesenchymal transition, colorectal cancer

## Abstract

**Simple Summary:**

Circulating tumor cell (CTC) detection is a non-invasive and easy way to collect and separate single cells to use as tumor biomarkers. In this article, we show the feasibility and clinical value of RNA sequencing of CTCs at the single-cell level by exploiting CTC-FIND with SMART-Seq v4 technologies in patients with metastatic colorectal cancer (mCRC). CTCs were classified by epithelial, epithelial-mesenchymal transition (EMT), and stem cell-related gene expression. A patient who had EMT gene expressing CTCs showed significantly shorter PFS and OS regardless of epithelial CTCs presence. Therefore, the presence of CTCs expressing EMT-related genes at the single-cell level may be ofprognostic value and a potential biomarker for risk stratification in patients with refractory mCRC who are considered to have highly heterogeneous features.

**Abstract:**

Background: Circulating tumor cells (CTCs) are a prognostic marker in patients with metastatic colorectal cancer (mCRC). However, little is known about the characterization of CTCs in mCRC at the single-cell level using RNA sequencing. The purpose of this study was to validate the capability to detect and isolate single CTCs for single-cell RNA sequencing (scRNA-seq) and to identify clinical significance at a single CTC level. Methods: Single CTCs from 27 mCRC patients were collected by CTC-FIND, which is comprised of filter separation and immunomagnetic depletion to collect ultra-pure CTC samples. To address tumor heterogeneity, CTCs were collected without relying on any traditional CTC markers, such as epithelial and mesenchymal cell antigens, and were undertaken by scRNA-seq using SMART-Seq v4. Results: We identified 59 single CTCs which were classified into four groups by epithelial, epithelial-mesenchymal transition (EMT) and stem cell-related gene expression. Patients receiving second or later-line treatment who had EMT gene expressing CTCs had a significantly shorter PFS and OS. Conclusions: Exploiting CTC-FIND with SMART-Seq v4 showed that scRNA-seq of CTCs may shed new insight into tumor heterogeneity of mCRC and that the presence of CTCs expressing EMT-related genes at the single-cell level could have prognostic value in mCRC patients.

## 1. Introduction

Colorectal cancer (CRC) is the third most common malignancy and the second leading cause of cancer-related deaths in the world [1]. Metastases are the main cause of CRC-related mortality and stem from a complex process involving multiple factors [2]. One of the reasons for this complexity is attributable to tumor heterogeneity referring to the differences in genetic and molecular characteristics and phenotypes between cancer cells within a single tumor or multiple metastatic sites [3]. Despite recent progress in using genome-wide and unbiased gene expression signatures for CRC subtype classification, such as the four consensus molecular subgroups (CMS) classification (CMS1: microsatellite instability, *BRAF* mutation, promoter hypermethylation, and immune infiltration. CMS2: activation of the Wnt and Myc pathways. CMS3: dysregulated metabolism and *KRAS* mutation. CMS4: mesenchymal and stroma-rich group associated with poor prognosis.), some CRC cases cannot be accommodated into a distinct CMS group due to a mixture of features observed in the other groups [4]. To understand these tumors, which are heterogeneously composed of different clones, it would be necessary to analyze and classify them at a single-cell level. However, invasive methods to address heterogeneity, i.e., tissue biopsies, still have many limitations, including patient risk, sample preparation, and difficulty in capturing inter-metastatic heterogeneity. The detection of circulating tumor cells (CTCs), which encompass the cell population that has shed into the vasculature from a primary and/or metastatic tumor site, represents a non-invasive and easy way to collect and separate single cells to address tumor heterogeneity. Thisholds great potential to overcome existing biopsy-related limitations [5].

RNA-sequencing (RNA-seq) technologies have been widely used for analyzing gene and transcript expression levels of bulk cells and had a pivotal role in the development of the CMS classification, which is associated with molecular features and clinical outcomes in CRC [6,7]. Furthermore, recently developed single-cell RNA sequencing (scRNA-seq) technologies provide a means for the analysis of any cell type at a single cell level and open the possibility to understand tumor heterogeneity unbiasedly using not only tumor tissue but also CTCs [8,9,10]. However, little is known about the characterization of CTCs in patients with metastatic colorectal cancer (mCRC) using scRNA-seq [11].

CTCs have been detected from peripheral blood in mCRC and their number has been associated with patient survival [12,13]. CTCs are, however, extraordinarily rare within the bloodstream and exhibit a phenotypic diversity, which is represented not only by the epithelial phenotype, but also by the epithelial-mesenchymal transition (EMT) and stem cell-like phenotypes [14]. Using the CellSearch^®^ system, which is the only method approved by the US Food and Drug Administration (FDA) at present, epithelial CTCs are detected in only 30–50% of mCRC patients [12,15,16]. Current marker-dependent CTC isolation and detection methods relying on the expression of epithelial antigens fail to capture the EMT and stem cell-like CTC phenotypes, which are essential for understanding such heterogeneity.

**Figure 1:**
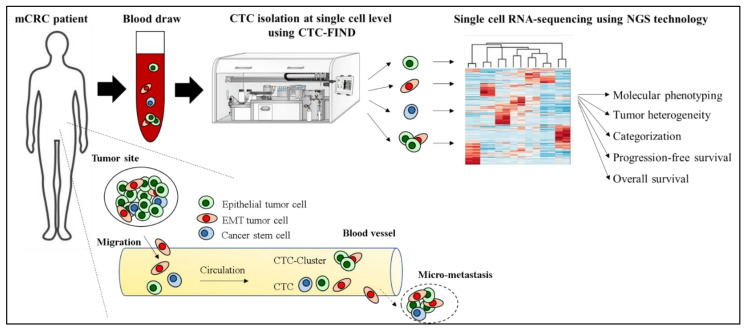
Hypothesis of addressing tumor heterogeneity and the clinical significance of CTCs in a mCRC patient at the single-cell level using CTC-FIND and RNA seq.

We recently developed CTC-FIND which is a technique combining filter separation with immunomagnetic depletion of CD45/50-expressing cells to isolate ultra-pure CTCs without relying on any traditional CTC markers, such as epithelial and mesenchymal cell antigens, and validated by blood samples collected from healthy volunteers [17]. By using CTC-FIND, we previously demonstrated that this method enabled us to not only collect typical epithelial cancer cells but also non-epithelial cancer cells with a residual of only about a hundred leucocytes from 8 mL whole blood. Furthermore, these samples could be used for gene testing [17]. We thus hypothesized that this method may be useful for isolating single CTCs of all phenotypes, such as epithelial, EMT and stem cell-like phenotypes, and may be able to address tumor heterogeneity and the clinical significance of CTCs in mCRC at the single-cell level using RNA seq (Figure 1).

The purpose of this study was to validate the capability to detect and isolate single CTCs for scRNA-seq by CTC-FIND and to identify molecular features and clinical significance at a single CTC level in patients with refractory mCRC who are considered to have highly heterogeneous features. We also established the clinical relevance of the presence of CTCs expressing EMT related genes at the single-cell level as a useful new prognostic marker in patients with mCRC.

## 2. Materials and Methods

### 2.1. Sample Collection

Patients were prospectively enrolled at the Norris Comprehensive Cancer Center, University of Southern California and at the Los Angeles County-University of Southern California Medical Center, between April 2019 and September 2019. A 10 mL whole blood sample was drawn from patients with mCRC (Table S1) candidates to receive systemic treatment (first and subsequent lines) before treatment start and collected into one Vacutainer K2 EDTA Tube (catalog no.: 368589; BD, Franklin Lakes, NJ, USA). 8 mL of blood for each sample was processed within an hour after drawing. All analyses were performed without knowledge of patients’ clinical status.

### 2.2. CTC Enrichment

CTC enrichment was performed as previously described [17]. A microfilter fabricated by nickel electroformation (catalog no.: AR229-150310-1; Optnics Precision Co., Ltd., Tochigi, Japan), was used for enriching CTCs. This microfilter had slit-shaped holes (6.5 × 88 μm) and a filtration area of 6 mm in diameter where about 20,000 holes were placed. Blood samples were loaded onto the reservoir of the filtration module and filtered by the tube pump. For removing residual erythrocytes, ammonium chloride solution (0.08%) was used after washing with phosphate-buffered saline containing EDTA (PBS-EDTA). To prevent a non-specific immune reaction and avidin-biotin binding of the remaining cells, PBS containing 0.2% bovine serum albumin (PBS-BSA) and avidin (catalog no.: sc-362068; Santa Cruz Biotechnology, Dallas, TX, USA) was loaded and incubated for 10 min. After washing with PBS, the cells were labeled with anti-human CD45 antibody (catalog no.: 14-0459-82; clone: HI30; Affymetrix, Santa Clara, CA, USA) and anti-human CD50 antibody (catalog no.: BMS111; Affymetrix, Santa Clara, CA, USA) in PBS-BSA with biotin medium (catalog no.: B0463; Tokyo Chemical Industry Co., Ltd., Tokyo, Japan) for 15 min, followed by washing with PBS. Then, the cells were labeled with a cocktail of secondary antibodies composed of anti-mouse IgG (F(ab’)2 specific) goat antibody labeled with Alexa 594 (catalog no.: 115-585-071; Jackson ImmunoResearch, West Grove, PA, USA) and anti-mouse IgG (Fc specific) goat antibody labeled with biotin (catalog no.: B7401; Sigma-Aldrich, St. Louis, MO, USA) in PBS-BSA containing Hoechst33342 for 15 min. After washing with PBS, the cells were mixed with neutralized avidin-coated magnetic beads (catalog no.: 03331; Bio-Adembeads StreptaDivin, 300 nm, Ademtech SA, Pessac, France) for 30 min. All processes were conducted on the microfilter.

### 2.3. Leukocyte Depletion

The leukocyte depletion was performed by using immunomagnetic negative selection as previously described [17]. The sample, once recovered from the microfilter, was loaded into a polyvinyl chloride tube (inner diameter 3.1 mm; Terumo, Tokyo, Japan) which was attached to a neodymium bar magnet (catalog no.: N40; 200 × 15 × 5 mm; NeoMag Co., Ltd., Tokyo, Japan) for 15 min without flow to attract leukocytes labeled with magnetic beads. The sample containing non-magnetic-labeled target cells was then collected from the tube outlet. The sample flow was controlled by the tube pump.

### 2.4. Isolating Single CTC Candidates

For isolating single cells, PBS-BSA was added to the sample depleted of leukocytes to reach a total volume of 3840 μL and divided into 384 Well Small Volume^TM^ LoBase Microplates (catalog no.: 788096; Greiner Bio-One GmbH, Frickenhausen, Germany). All microwells were observed using a fluorescence microscope (IX 73; Olympus Corp., Japan (Ex. 570–590 nm and Em. 610–640 nm for Alexa 594, Ex. 360–370 nm and Em. 450–490 nm for Hoechst 33342). The nuclei (+)/CD45/50- Alexa 594 (−) cells with cell-like morphology were identified as CTC candidates. In the case of two or more single cells in a well, with the exception of clustered cells, the sample was divided again into other microwells until isolated to a single cell.

### 2.5. Single Cell RNA Sequencing and Analysis

Total RNA was extracted from intact single cells, amplified to cDNA transcripts and purified using SMART-Seq^®^ v4 Ultra^®^ Low Input RNA Kit for Sequencing (catalog no.: 634893; Takara Bio USA, Inc., Mountain View, CA, USA). cDNA concentration was measured with Nanodrop (ThermoFisher Scientific, Waltham, MA, USA). cDNA libraries for RNA sequencing were constructed from Nextera XT DNA Library Preparation Kit (catalog no.:FC-131-1096; Illumina Way, San Diego, CA, USA). Before next-generation sequencing, the libraries’ concentrations were measured with Qubit dsDNA HS assay and size distribution was checked with a bioanalyzer. Libraries then underwent sequencing to 600 million reads of 150 base-pair lengths, paired-end using HiSeq4000 system. The reads were first mapped to the latest UCSC transcript set using Bowtie2 version 2.1.0 (RRID:SCR_016368) and the relative gene expression was quantified as transcript per million (TPM) using R RSEM v1.2.15 (RRID:SCR_013027). Sequencing and analysis were conducted by Quickbiology (www.quickbiology.com, accessed on 14 June 2019 and 15 October 2019, Pasadena, CA, USA). To explore the associations between cell groups, we performed hierarchical clustering by using TPM of each gene set (Epithelial cell, Leucocyte, Endothelial cell, Stem cell and EMT markers). Hierarchical clustering and heatmap visualization were performed by R language version 3.6.2 (R Foundation for Statistical Computing, Vienna, Austria) and Microsoft Excel 2016 software (RRID:SCR_016137, Microsoft, Redmond, WA, USA).

### 2.6. Statistical Analysis

All analyses were performed using the BellCurve for Excel (Social Survey Research Information Co., Ltd., Tokyo, Japan). Fisher’s exact test, Chi-square test and residual analysis were used to assess statistically significant differences between CTC groups. Progression-free survival (PFS) and overall survival (OS) were estimated by the Kaplan–Meier method and calculated using the Generalized Wilcoxon test. PFS was defined as the interval between the date of treatment start and the date of confirmed disease progression or death. OS was defined as the interval between the date of treatment start and the date of death. Data of patients without disease progression or death were censored at the date of the last follow up. A *p*-value of < 0.05 was considered statistically significant.

## 3. Results

### 3.1. Detection and Isolation of CTC Candidates from mCRC Patients

**Figure 2:**
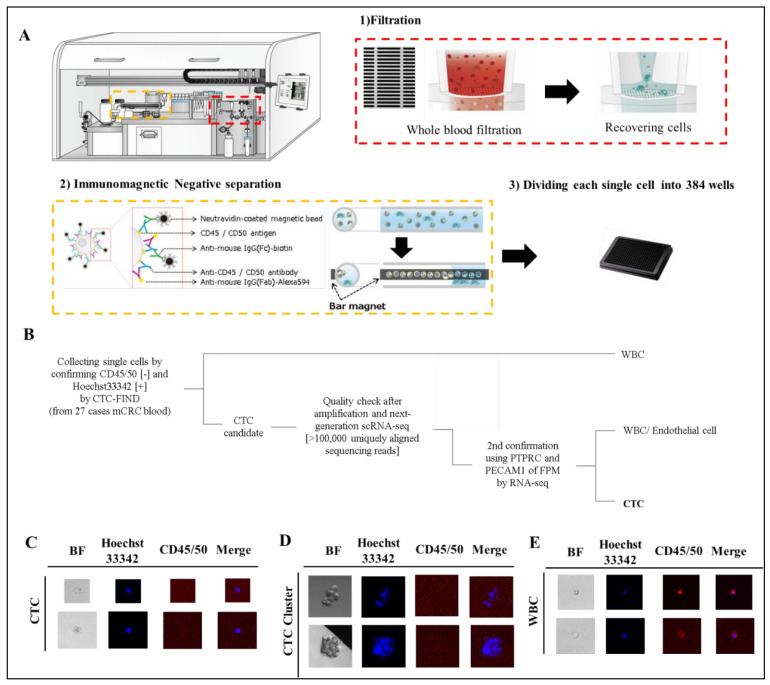
Separation of CTCs and leukocyte or endothelial cells. (**A**) Technical concept using CTC-FIND for single cell isolation. (**B**) Scheme of cell classification. (**C**–**E**) Representative fluorescence image of CTCs detected in clinical blood specimens. The blood samples (8 mL) collected from patients with mCRC were evaluated by fluorescence microscopy after separation to single cells by CTC-FIND. Specimens were enriched and stained with Hoechst 33342 (pseudo colored blue) and negative markers composed of anti-CD45 and anit-CD50 antibodies (pseudo colored red), followed by the acquisition of fluorescence images for each marker and digital overlay of the two images (merge). Hoechst 33342-positive and CD45/50-negative CTCs were counted and collected from each specimen. Gallery of CTCs (**C**), CTC clusters (**D**) and leucocytes (**E**). Original magnification, ×500.

We collected blood samples from 27 patients with mCRC (Table S1) before the start of a new treatment line. 110 cells and cell-clusters, which were CD45/50 negative and Hoechst33342 positive, were identified as CTC and CTC-cluster candidates by fluorescence observation (Figure 2B–E). All cells were processed for single-cell RNA sequencing by SMART-Seq v4 techniqueand read using HiSeq4000 system. All samples had RNA of sufficient quality for amplification and next-generation RNA sequencing [>100,000 uniquely aligned sequencing reads] [18], and of these, 109 cells (99%) had >400,000 uniquely mapping reads. The gene expression of each cell was quantified as TPM. Fifty-nine cells and cell-clusters (54% of collected cells) were identified as CTCs and CTC-clusters, although some of the cells were eliminated as leukocytes, endothelial cells or CTC clusters with mixed leukocytes by checking leukocyte marker and endothelial cell marker (Figure 2A). Eventually, we identified 59 single CTCs and CTC clusters from 24 patients in total for this study (detection 89%, count range 1–9, median = 2 per case).

### 3.2. Epithelial Phenotyping of Single CTCs and CTC-Clusters

**Figure 3:**
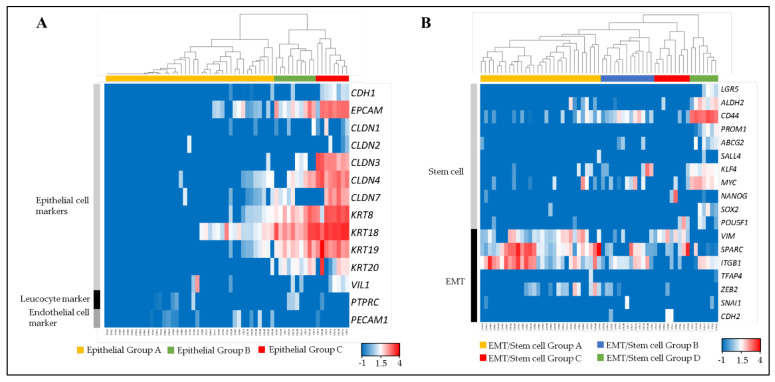
Molecular phenotyping of single CTCs and CTC-clusters. The dendrogram shows cell type clusters and the heatmap shows the log10 (FPM) values of cell-type markers. (**A**) Heatmap showing the raw log10 (FPM) values for known markers of epithelial cells (*CDH1*, *EPCAM*, *CLDN1*, *CLDN2*, *CLDN3*, *CLDN4*, *CLDN7*, *KRT8*, *KRT18*, *KRT19*, *KRT20* and *VIL1*), leukocytes (*PTPRC*), and endothelial cells (*PECAM1*). (**B**) Heatmap showing the raw log10 (FPM) values for known markers of stem cells (*LGR5*, *ALDH2*, *CD44*, *PROM1*, *ABCB2*, *SALL4*, *KLF4*, *MYC*, *NANOG*, *SOX2* and *POU5F1*) and EMT (*VIM*, *SPARC*, *ITGB1*, *TFAP4*, *ZEB2*, *SNAI1* and *CDH2*).

Fifty-nine single CTCs and CTC-clusters were divided into three groups (Epithelial Group A, B and C) by clustering analysis, using 12 epithelial cell markers (*CDH1*, *EPCAM*, *CLDN1*, *CLDN2*, *CLDN3*, *CLDN4*, *CLDN7*, *KRT8*, *KRT18*, *KRT19*, *KRT20* and *VIL1*) [19,20,21,22], a leucocyte marker (*PTPRC*), and an endothelial cell marker (*PECAM1*) (Figure 3A).

Epithelial Group A comprised the cells that expressed low or non-epithelial cell markers. Epithelial Group B comprised middle-epithelial marker expression. The cells in this group mainly expressed *EPCAM*, *CLDN4*, *KRT8*, *KRT 18*, *KRT 19*, and *KRT 20* as epithelial markers. The epithelial gene expression score of this group was significantly higher than Epithelial Group A (Figure S1A). Epithelial Group C comprised high-epithelial marker expression adding *CLDN3* and *CLDN7* to Group B. This group had a significantly higher epithelial gene expression score than Epithelial Groups A and B.

### 3.3. EMT and Stem Cell Related Phenotyping

All single CTCs and CTC-clusters were also divided into four groups (EMT/Stem cell Group A, B, C and D) by using 11 stem cell related markers (*LGR5*, *ALDH2*, *CD44*, *PROM1*, *ABCB2*, *SALL4*, *KLF4*, *MYC*, *NANOG*, *SOX2* and *POU5F1*) and seven EMT related markers (*VIM*, *SPARC*, *ITGB1*, *TFAP4*, *ZEB2*, *SNAI1* and *CDH2*) (Figure 3B). These markers were selected from genes that were previously reported as stem cell or EMT-related genes in colorectal cancer cells [23,24,25,26,27,28,29,30,31,32,33,34,35,36,37,38,39,40]. EMT/Stem cell Group A had a significantly higher EMT-related gene expression score than other groups, mainly contributed to by *VIM*, *SPARC* and *ITGB1*, but expressed low stem cell related markers (Figure S1B,C). EMT/Stem cell Group B had a significantly higher stem cell related gene expression score than EMT/Stem cell Group A by expressing *CD44*, *KLF4* and *MYC*, but significantly lower than EMT/Stem cell Group D. EMT/Stem cell Group C had significantly lower stem cell related markers expression than EMT/Stem cell Group A and also a significantly lower EMT-related gene expression score than EMT/Stem cell Group D. EMT/Stem cell Group D, which strongly expressed *CD44*, *ALDH2* and *MYC*, had significantly higher stem cell related marker expression than others, but the lowest EMT-related gene expression score. In summary, EMT/Stem cell Group A showed high stem-like/low EMT, EMT/Stem cell Group B showed low stem-like/middle EMT, EMT/Stem cell Group C showed low stem-like/low EMT, and EMT/Stem cell Group D showed low stem-like/high EMT.

### 3.4. Categorization of CTC Phenotypes

Each CTC and CTC-cluster, furthermore, was separated into 12 categories based on the three Epithelial Groups and four EMT/Stem cell Groups represented in Figure 3 (Table 1). A statistically significant correlation between epithelial-cell subtypes and stem/EMT-related subtypes was found in four categories by the chi-square test and residual analysis. Category 1 to 4 were: high epithelial and high stem-like/low EMT, low epithelial and low stem-like/middle EMT, middle epithelial and low stem-like/low EMT, and low epithelial and low stem-like/high EMT, respectively. Notably, no cells fell into the category of low epithelial and high stem-like/low EMT and high epithelial and low stem-like/high EMT (significant *p*-value).

### 3.5. Phenotypic Heterogeneity of CTCs in mCRC

We examined the heterogeneity of CTCs within every single patient using the four significant categories identified in Table 1 (detailed in the previous paragraph) plus an additional category, “others,” comprising all CTCs outside of Group 1 to 4 (Figure S2). Thirteen cases had two or more categorized CTCs and CTC clusters and 46% of them had a combination of Category 2 and 4 which represent middle or high EMT but low epithelial. One case had Category 3 which is middle epithelial and Category 4 which is high EMT, only. 

These results show that CTCs from patients with mCRC display tumor heterogeneity, mainly a variation of EMT-related gene expression, at the single-cell level.

### 3.6. Phenotypic Frequency

We further compared the positive rates in the case of selection by epithelial, EMT and epithelial and/or EMT CTCs (Table 2). Patients who had one or more each epithelial, EMT and epithelial/EMT CTCs were counted as a positive of each type of CTC. EMT CTCs were detected in 74% of patients (*n* = 20), even though epithelial CTCs were found only in 30% (*n* = 8). Notably, 89% of patients were CTC-positive by combining epithelial and/or EMT CTCs (*n* = 24). There were significant differences in positive rates between epithelial CTCs and EMT CTCs (*p* = 0.0024), as well as between epithelial CTCs and epithelial/EMT CTCs (*p* = 0.0001).

### 3.7. Prognostic Relevance of CTC Phenotypes

We first examined the prognostic relevance of CTC Category 1 to 4. Kaplan–Meier plots of PFS and OS of 27 mCRC cases showed no significant differences between Category 1 to 4 and others respectively, although Category 2 and 4 which are middle or high EMT but low epithelial had a tendency for shorter PFS and OS (Table S2). 

**Figure 4:**
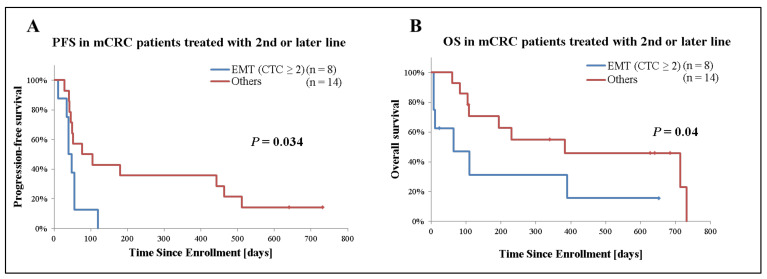
Kaplan–Meier plots of 22 mCRC cases receiving second- or later-line treatment stratified by presence of EMT CTCs. (**A**) Analysis of progression-free survival (PFS), (**B**) overall survival (OS). Patient receiving second- or later-line of treatment were grouped based on the presence and number of EMT CTCs. Patients who only had detectable EMT CTCs and 2 or more CTCs were categorized as “EMT (CTC ≥ 2)”. ‘Others’ indicates patients with <2 EMT CTCs or no detectable CTCs. *p*-values were calculated with the Generalized Wilcoxon test and are shown in the graph.

Secondly, we analyzed the prognostic significance of EMT CTCs in patients with mCRC receiving second- or later-line treatment (*n* = 22). Among patients who only had detectable EMT CTCs, those with 2 or more CTCs (*n* = 8) had a significantly inferior median PFS (40 days [95%CI: 28–52] versus 76 days [95%CI: −20–172], *p* = 0.034) and median OS (64 days [95%CI: −47–175] versus 383 days [95%CI: −139–905], *p* = 0.04) compared with those with 1 or none. On the other hand, no significant difference was observed based on epithelial CTCs presence (Figure 4 and Table 3).

## 4. Discussion

Several techniques have been established to accurately detect CTCs and CTC clusters in blood, and growing evidence is accumulating on single-cell analysis of CTCs as a means to provide critical insights into tumor progression and metastases [41,42]. CTC count has been shown to have a significant prognostic impact in CRC and CTCs are considered promising biomarkers for the management of this malignancy both in the adjuvant (patient stratification based on the risk of recurrence, minimal residual disease evaluation) and in the metastatic setting (monitoring of systemic therapy, detection of therapy resistance) [43]. Molecular characterization of detected CTCs holds an even greater potential to address tumor heterogeneity, dynamic changes under treatment pressure, and metastatic spread. Only a few studies, however, have been published on the bulk RNA sequencing analysis in CTCs [8,44], and the characterization of CTCs from CRC is yet unknown at the single-cell level. Furthermore, commonly used marker-based detection of CTCs fails to encompass CTCs phenotypic diversity. Here, we present a novel approach to exploit single CTCs for scRNA-seq by combining CTC-FIND and SMART-Seq v4 technique. The present study is, to our knowledge, the first to characterize CTCs from patients with mCRC at the single-cell level evaluating distinct CTCs phenotypes and their prognostic impact.

Before the phenotyping of patient samples, we have validated scRNA-seq using healthy blood samples spiked with human colorectal cancer cell lines (Figure S3). We were able to separate colorectal cancer cell lines and WBC clearly by using epithelial cells, leukocytes, endothelial cells, EMT and stem cells markers. Although we collected 110 cells and cell-clusters as CTC candidates from 27 cases of mCRC which were CD45/50 negative and Hoechst33342 positive; 51 cells (46%) were eliminated as leukocytes, endothelial cells, or CTC clusters with mixed leukocytes after analyzing scRNA-seq, due to the expression of leukocyte markers (*PTPRC*) or endothelial cell markers (*PECAM1*). This result suggests that detecting leukocyte markers by immunofluorescent staining and by RNA-seq have different sensitivities, and only using immunofluorescent staining could lead to the selection of cells that are false negative for leukocyte markers. On the other hand, this result also suggests that there is a technical limitation that does not allow for the discrimination between CTC clusters conjugated leukocytes and leukocytes alone, potentially causing some amounts of CTCs and CTC clusters to be missed.

Molecular phenotyping of single CTCs and CTC clusters revealed that epithelial CTCs express *CLDN3*, *CLDN4* and *CLDN7*, which are known to be strongly expressed in primary and metastatic CRC [19], in addition to *EPCAM*, *KRT8*, *KRT 18*, *KRT 19* and *KRT20,* which were recognized as CTC markers in mCRC [20]. Interestingly, CTCs of Category 1, which express stem-like cell markers, such as *CD44*, *ALDH2* and *MYC*, co-expressed epithelial markers (Table 1). Moreover, all the CTC clusters collected in this study were classified into the high epithelial and high stem-like cell marker-expressing group. Since a CTC cluster is defined as two or more conjunct CTCs, *CLDN3*, *CLDN4* and *CLDN7*, which are members of the claudin family considered to comprise the major component of tight junctions, could be more strongly expressed than in single CTCs which do not connect with any other cells. Furthermore, since several studies have reported that cancer stem-like cells are subpopulations in the tumor that are endowed with the ability to self-renew and differentiate into non–stem cancer cells that comprise the bulk of the tumor [45,46], it might be reasonable to assume that CTC clusters can also include subpopulations and consist of stem-like cells as well as epithelial cells.

On the other hand, EMT-phenotype CTCs that express *VIM*, *SPARC* and *ITGB1*, expressed no or low epithelial markers. These results suggest that epithelial and EMT CTCs have contrasting features in terms of epithelial and EMT-related gene expression, and the traditional CTC detecting methods based on anti-EpCAM antibodies could fail to collect most EMT CTCs. Since blood cells are a type of mesenchymal cell, EMT CTCs cannot be selected by mesenchymal cell markers when purified from blood samples. Therefore, our findings suggest that CTC-FIND, which is a technique combining filter separation with immunomagnetic depletion of CD45/50-expressing cells without relying on any CTC markers, is suitable for collecting EMT CTCs as well as epithelial CTCs. In addition to *VIM*, commonly used for detecting the EMT phenotype, molecular phenotyping revealed that EMT CTCs expressed *SPARC* and *ITGB1* genes. ITGB1 is an integrin family member and known as a membrane receptor involved in cell adhesion and recognition in a variety of processes including metastatic diffusion of tumor cells. It has also been reported that ITGB1 regulates the growth and apoptosis of human CRC cells [47]. SPARC is a well-known ECM protein gene and it has also been reported that the SPARC protein increases cancer cell invasiveness and migration [48]. These findings suggest that *SPARC* and *ITGB1* could be potential EMT CTC markers in addition to *VIM*. Although molecular phenotyping was undertaken using well-studied epithelial, EMT and stem-related genes, there is a technical limitation that the combination of each gene set for molecular phenotyping has not been validated yet. In future studies, we strive to evaluate this method not only for CTCs but also tissue samples in larger patient numbers so that gene sets and classification can be wellvalidated. Nevertheless, these results suggest that the gene set used in this study is capable of CTC phenotyping in patients with mCRC.

EMT CTCs were detected in 74% while epithelial CTCs were found in only 30% of patients with mCRC in this study. A previous study with the conventional CTC detection system using anti-EpCAM and -CK antibody has reported that 30–50% of patients with mCRC had epithelial CTCs [12,15,16]. The capability of our method to detect epithelial CTCs is thought to be equivalent to the conventional method, although data from different studies cannot be directly compared. As reported in various studies [49,50], this result also supports the evidence that EMT CTCs are found more frequently than epithelial CTCs, and it is essential to implement EMT CTCs detection in addition to epithelial CTCs for high-sensitivity CTC detection in patients with mCRC. Additionally, in our series patients receiving second- or later-line treatment who had two or more middle or high EMT gene expressing CTCs had a significantly shorter PFS (*p* = 0.034) and OS (*p* = 0.04) regardless of epithelial CTCs presence. Patients treated with first-line therapy were excluded from our exploratory survival analysis in order to minimize survival and outcome differences linked to differential treatment benefit observed in first- versus subsequent treatment lines. In fact, patients treated with first-line therapy had longer PFS and OS than patients who received later-line therapy regardless of EMT or epithelial CTCs detection (data not shown). Limitations of our analysis are the small patient number and patient heterogeneity, however, these results suggest that the CTC EMT phenotype is associated with poor prognosis in patients with mCRC.

## 5. Conclusions

Our strategy exploiting CTC-FIND with SMART-Seq v4 technologies demonstrates the feasibility and clinical value of RNA sequencing of CTCs at the single-cell level in patients with mCRC. This study shows that epithelial and EMT CTCs have contrasting features in terms of epithelial and EMT-related gene expression, and CTC clusters co-express epithelial and stem-like cell markers. Moreover, *SPARC* and *ITGB1* could be potential EMT CTCs markers. In addition, we reported that CTCs with an EMT phenotype are detected more frequently than epithelial CTCs and are associated with poor prognosis in patients with mCRC. Therefore, the presence of CTCs expressing EMT-related genes at the single-cell level could have prognostic value in patients with mCRC.

## Figures and Tables

**Table 1:** Correlation between epithelial-cell subtypes and stem/EMT-related subtypes by Chi-square test and residual analysis.

	EMT/Stem Group A:	EMT/Stem Group B:	EMT/Stem Group C:	EMT/Stem Group D:
	High Stem/Low EMT	Low Stem/Middle EMT	Low Stem/Low EMT	Low Stem/High EMT
Epithelial Group A: High	7	0	1	0
	↑ **	ns	ns	↓ **
Epithelial Group B: Middle	0	0	7	3
	ns	ns	↑ **	ns
Epithelial Group C: Low	0	9	4	28
	↓ **	↑ **	↓ **	↑ **

↑ **: *p* < 0.01 (Significantly high); ↑ **: *p* < 0.05; ↓ **: *p* < 0.01 (Significantly Low); ↓ **: *p* < 0.05; ns: non-significant.

**Table 2:** Comparison of positive rates in case of selected by epithelial, EMT and epithelial and/or EMT CTCs.

Type of CTC	Patient #		Detection Rate
	Positive	Negative	
Epitherial	8	19	30% ^**,***^
EMT	20	7	74% ^**^
Epitherial/EMT	24	3	89% ^***^

^**^, ^***^ Show significant differences (*p* < 0.01) by Fisher’s exact test, ^**^ between the first two values and ^***^ between the first and last values. # = patient number.

**Table 3:** PFS and OS in patients with mCRC receiving second- or later-line of therapy.

CTC Type	CTC #	*n*	PFS			OS		
			Median	(95% CI), Days	*p*-Value	Median	(95% CI), Days	*p*-Value
EMT	≥1				0.075			0.212
−		8	180	(−272, 632)		230	(−306, 766)	
+		14	48	(43, 53)		110	(38, 182)	
EMT	≥2				0.034 *			0.04 *
−		14	76	(−20, 172)		383	(−139, 905)	
+		8	40	(28, 52)		64	(−47, 175)	
Epithelial	≥1				0.825			0.496
−		20	52	(42, 62)		230	(−116, 577)	
+		2	41	-		194	-	
Epithelial	≥2				0.336			0.327
−		21	55	(45, 65)		194	(−29, 359)	
+		1	464	-		-	-	
Epithelial/EMT	≥1				0.643			0.74
−		17	52	(43, 61)		230	(−232, 692)	
+		5	105	(32, 242)		194	(2, 385)	
Epithelial/EMT	≥2				0.347			0.685
−		18	49	(40, 57)		230	(−94, 554)	
+		4	105	(−265, 475)		105	(105, 105)	

* Significant difference (*p* < 0.05) by the Generalized Wilcoxon test. **#** = number.

## Data Availability

The RNA-seq reads are available through the NCBI’s Sequence Read Archive (SRA) under the accession number PRJNA759644 (https://www.ncbi.nlm.nih.gov/sra, accessed on 28 September 2020).

## Supplementary Materials

The following are available online at https://www.mdpi.com/article/10.3390/cancers13194862/s1, Figure S1: Gene Expression analysis of CTC groups clustered by molecular phenotyping (Heatmap analysis, Figure 2), Figure S2: CTCs count and sub-groups from 27 mCRC cases, Figure S3: Molecular phenotyping of single human colorectal tumor cell lines HT29, SW480 and SW620 as well as WBC, Table S1: Baseline patient characteristics (*n* = 27), Table S2: PFS and OS in patients with mCRC classified by CTC categories.
