# Supplementary material for: Clinical Significance of Circulating Tumor Cell Induced Epithelial-Mesenchymal Transition in Patients with Metastatic Colorectal Cancer by Single-Cell RNA-Sequencing
Source: Cancers (Basel). 2021 Sep 28;13(19):4862. doi: 10.3390/cancers13194862 (PMC8507666; doi:10.3390/cancers13194862)
Supplement: Supplementary file 1 [file cancers-13-04862-s001.zip › cancers-1372570-supplementary.pdf]

Supplemental Figure legend

Figure S1. Gene Expression analysis of CTC groups clustered by molecular phenotyping (Heatmap analysis, Figure 2). Each group shows single CTC's gene expression as a score, calculated using the mean value of the expression (FPK) of each gene for a selected pathway. (A) shows scores of Epithelial gene expressions, as according to Epithelial group A to C by Heatmap analysis. (B) and (C) show scores of Stem cell related and EMT-related gene expressions, respectively, according to Epithelial group A to C by Heatmap analysis. *P*-values were calculated based on Mann-Whitney U test, where * denotes $P < 0.05$, ** denotes $P < 0.01$.

Supplemental Figure

Figure S1.

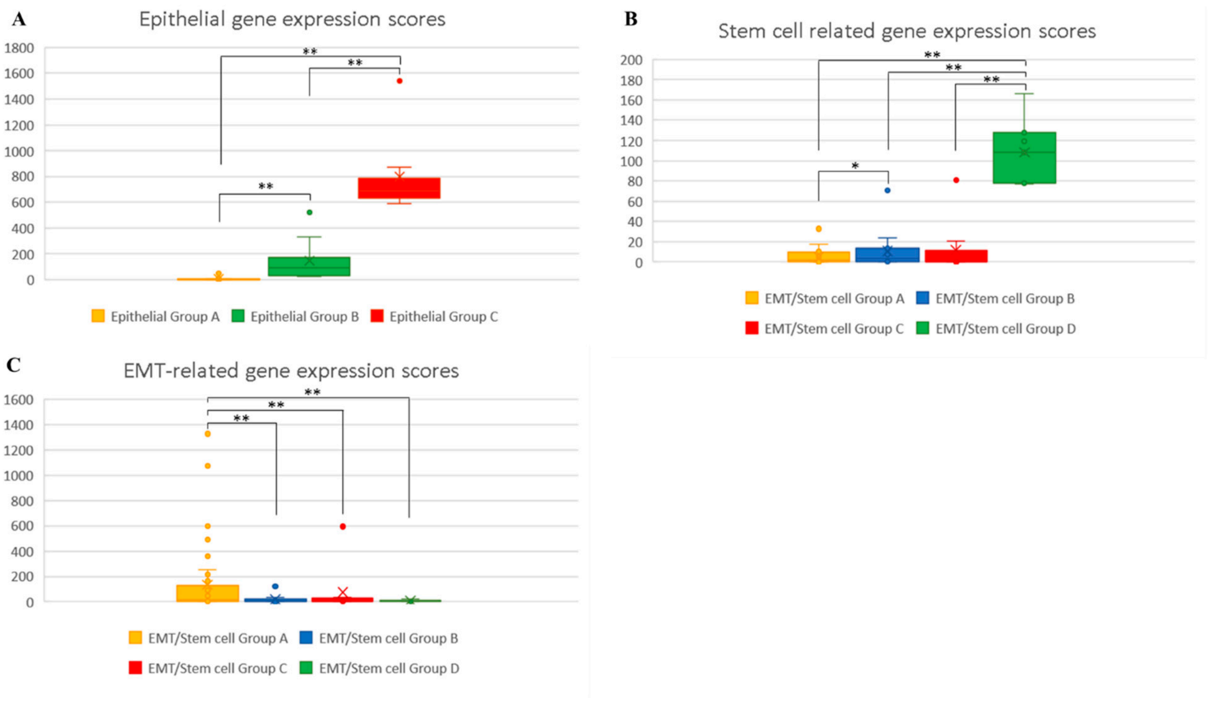

Supplemental Figure legend

Figure S2. CTCs count and sub-groups from 27 mCRC cases. Each single CTC and CTC cluster are classified into 5 groups (Group 1 to 4 and others) which were identified by correlation between Epithelial-cell subtypes and Stem/EMT-related subtypes as shown in table 1. Category 1: high Epithelial, high Stem-like and low EMT. Category 2: low Epithelial, low Stem-like and middle EMT. Category 3: middle Epithelial, low Stem-like and low EMT. Category 4: low Epithelial, low Stem-like and high EMT. 'Others' includes the infrequent CTCs which were categorized outside of Group 1 to 4. A CTC cluster was counted as one even if containing several CTCs.

Supplemental Figure

Figure S2.

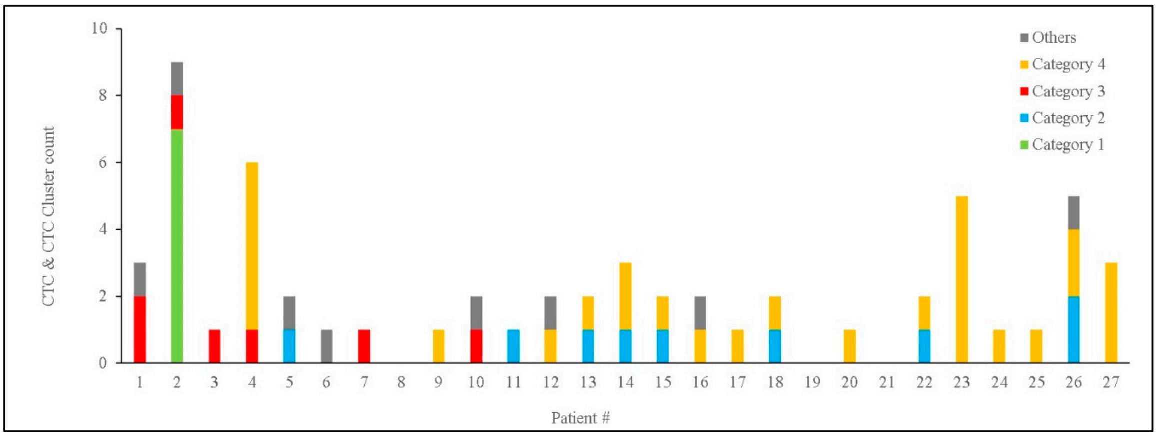

Supplementary Material and Methods

1. Cell culture and validation of scRNA-seq using cell lines

The human colorectal tumor cell lines HT29, SW480 and SW620 were cultured in accordance with the specifications supplied by the American Type Culture Collection. All cells were grown in a culture dish to sub-confluence prior to each experiment and collected using 0.01% EDTA and 0.125% trypsin. Then the collected cells were then washed with phosphate-buffered saline (PBS). The number of cells was measured using a coulter counter (MoxiZ; ORFLO Technologies, Hailey, ID, USA).

Approximately 200 cells of cell line were spiked into 8 mL of whole blood collected using vacutainer tubes containing the anti-coagulant EDTA. The target cell enrichment,

leukocyte depletion, isolating target cells and single cell RNA Sequencing has been conducted by the same procedure described in Material and Methods.

Supplemental Figure legend

Figure S3. Molecular phenotyping of single human colorectal tumor cell lines HT29, SW480 and SW620 as well as WBC. The heatmap shows the log10 (FPM) values of epithelial cells, leucocytes, endothelial cells and stem cells markers.

Supplemental Figure

Figure S3.

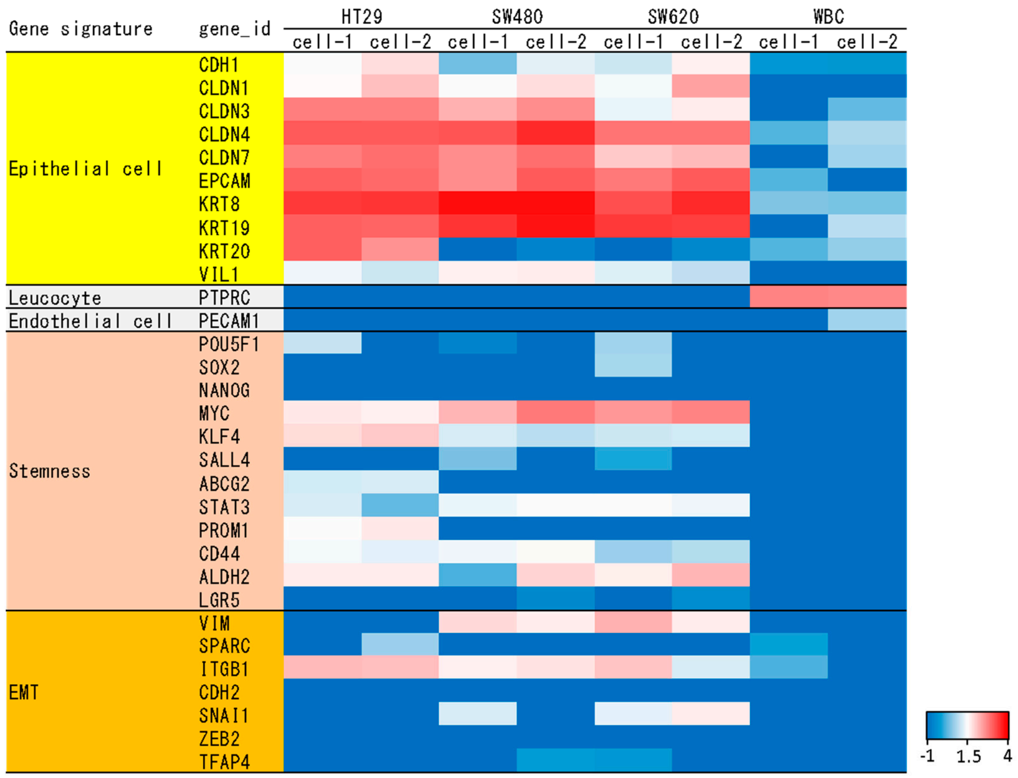

Supplemental Table

Table S1.

Baseline patient characteristics (n=27)

Characteristic	n (%)
Age, years	
Median (range)	52 (22-72)
Sex	
Male	19 (70.4)
Female	8 (29.6)
Race	
White	13 (48.1)
African American	1 (3.7)
Asian	4 (14.8)
Hispanic	8 (29.6)
Other	1 (3.7)
Line of therapy	
1st	5 (18.5)
2nd	11 (40.7)
3rd	6 (22.2)
4th	2 (7.4)
5th or more	3 (11.1)

Supplemental Table

Table S2.

PFS and OS in patients with mCRC classified by CTC categories.

CTC type	n	PFS			OS		
		Median	(95% CI), days	*P*-value	Median	(95% CI), days	*P*-value
Category 1				0.577			0.29
−	26	56	(5, 108)		382	(93, 673)	
+	1	437	−		−	−	
Category 2				0.128			0.291
−	19	105	(-71, 281)		383	(-310, 1076)	
+	8	48	(27, 69)		105	(-254, 474)	
Category 3				0.709			0.514
−	21	56	(20, 92)		383	(19, 747)	
+	6	105	(-343, 553)		194	(-363, 751)	
Category 4				0.319			0.383
−	11	180	(-196, 556)		700	(66, 1334)	
+	16	49	(41, 57)		383	(61, 705)	

Category 1: high Epithelial, high Stem-like and low EMT subtype.

Category 2: low Epithelial, low Stem-like and middle EMT subtype.

Category 3: middle Epithelial, low Stem-like and low EMT subtype.

Category 4: low Epithelial, low Stem-like and high EMT subtype.

P-values by Generalized Wilcoxon test.
